# Sex differences in spatial learning and memory and hippocampal long-term potentiation at perforant pathway-dentate gyrus (PP-DG) synapses in Wistar rats

**DOI:** 10.1186/s12993-021-00184-y

**Published:** 2021-11-01

**Authors:** Samaneh Safari, Nesa Ahmadi, Reihaneh Mohammadkhani, Reza Ghahremani, Maryam Khajvand-Abedeni, Siamak Shahidi, Alireza Komaki, Iraj Salehi, Seyed Asaad Karimi

**Affiliations:** 1 Neurophysiology Research Center, Hamadan University of Medical Sciences, Hamadan, Iran; 2 Department of Exercise Physiology, Faculty of Sport Sciences, University of Birjand, Birjand, Iran; 3 Department of Neuroscience, School of Science and Advanced Technologies in Medicine, Hamadan University of Medical Sciences, Shahid Fahmideh Street, Hamadan, Iran

**Keywords:** Long-term potentiation, Hippocampus, Dentate Gyrus, Spatial learning and memory, Sex difference, Wistar Rat

## Abstract

**Background:**

Recent studies show that gender may have a significant impact on brain functions. However, the reports of sex effects on spatial ability and synaptic plasticity in rodents are divergent and controversial. Here spatial learning and memory was measured in male and female rats by using Morris water maze (MWM) task. Moreover, to assess sex difference in hippocampal synaptic plasticity we examined hippocampal long-term potentiation (LTP) at perforant pathway-dentate gyrus (PP-DG) synapses.

**Results:**

In MWM task, male rats outperformed female rats, as they had significantly shorter swim distance and escape latency to find the hidden platform during training days. During spatial reference memory test, female rats spent less time and traveled less distance in the target zone. Male rats also had larger LTP at PP-DG synapses, which was evident in the high magnitude of population spike (PS) potentiation and the field excitatory post synaptic potentials (fEPSP) slope.

**Conclusions:**

Taken together, our results suggest that sex differences in the LTP at PP-DG synapses, possibly contribute to the observed sex difference in spatial learning and memory.

## Background

Male and female nervous system respond differently to abnormal physiological situations [1], so finding sex differences in diverse brain functions seems essential. Studies have shown that gender may have a substantial effect on human cognitive ability [2]. Men and women appear to have different strategies for decision-making and memory encoding. Because the nervous system controls cognitive behaviour, these sex-related functional differences may be associated to the sex-specific structure of the neuronal circuits in the nervous system [3].

Spatial memory is responsible for spatial orientation and retrieving information about the locations of objects and places in the environment [4]. Gender differences in cognition have been described in cognitive and behavioral psychology for many years [5]. Moreover, several studies have reported sex differences in spatial ability in humans, primates, and rodents [4, 6–10]. Overall, males excel in spatial processing, while females excel in verbal work [11–14]. Also. studies on rodents and humans have shown that males have better performance on spatial memory tasks to females [4, 15–17].

On the other hand, some groups claim that there is no gender difference in spatial memory ability [18–20]. According to the literature, the results seem to be varied and divisive, from ‘‘substantial difference” to ‘‘no significant difference”. In the current work, we first attempted to determine sex differences in spatial memory and sought to find possible underlying mechanisms in rats.

Some works have shown that the brain areas related with spatial anility can be different in each sex or have different functions [21–25]. Nevertheless, the main neural circuits and underlying mechanisms responsible for these differences are not fully understood and there are contradictions in this regard.

There is much evidence that the hippocampus plays an important role for long-term memory and is necessary for episodic and spatial memory [26–28]. The hippocampus shows high degree of synaptic plasticity. Two main classes of synaptic plasticity are long-term potentiation (LTP) and long-term depression (LTD). It is well well-known that hippocampal LTP is the essential mechanism for spatial memory [29, 30]. On the other hand, due to the main role of LTP in memory, differences in the induction or expression of LTP between males and females may be the cause of gender differences in spatial memory.

There is controversy about sex differences in synaptic plasticity. Strong sex differences in LTP induction have been reported in dentate gyrus (DG) of pentobarbital and/or urethane-anesthetized rats [31, 32] and also in CA1 region of hippocampal slices [33–35]. However, some studies have reported no difference in LTP at the perforant pathway (PP)-DG synapses [36, 37]. It has been reported that the LTP magnitude is not different depending on the sex [35, 37]. Stephen et al. have reported sex differences in hippocampal LTP, but their results showed that, unlike sex difference in field excitatory post synaptic potentials (fEPSP) slope LTP, a sex difference in population spike (PS) amplitude LTP was not apparent [32]. Monfort et al. have also shown that hippocampal LTP in the CA1 region is reduced in mature compared to young male rats but not in female rats [38]. In our opinion, these findings should be repeated because there is no consistency in the literature about sex differences in cognitive functions such as spatial learning and memory and LTP. Therefore, the aim of the current work was to evaluate whether is there a difference between male and female rats in spatial learning and memory in the Morris water maze (MWM)? And whether changes in spatial learning and memory are related to hippocampal LTP at PP-DG synapses.

## Results

### Behavior

#### Memory acquisition in MWM

**Fig. 1:**
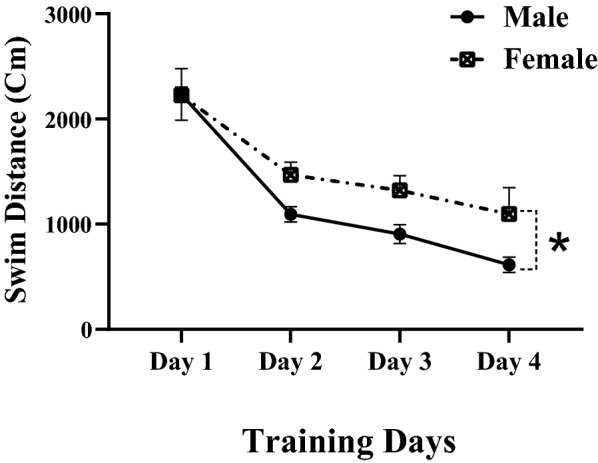
Swim traveled to find the hidden platform during training days. Each point represents the daily average of each group. Performance of all animals improved during the 4-day training period. Swim distance in training days decreased in both male and female rats. Male rats had shorter swimming paths to escape onto the hidden platform than female rats. Data presented as mean ± S.E.M. *p < 0.05

**Fig. 2:**
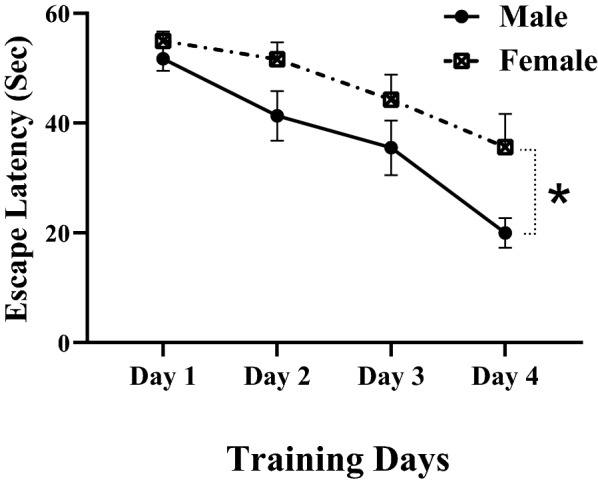
Latency to find a hidden platform during training days. Each point represents the daily average of each group. Performance of all animals improved during the 4-day training period. The time to locate the hidden platform decreased in both male and female rats. Male rats had less latency to escape onto the hidden platform than female rats. Data presented as mean ± S.E.M. *p < 0.05

The time to locate the hidden platform or swim distance in training days decreased in both male and female rats. This means that the performance of animals improved during the 4-day training period. Figure 1 shows the data for the swimming distance to find the hidden platform. There was a significant main effect of training day on swim distance [F (1.623, 22.72) = 56.59, P < 0.0001]. In general, male rats had shorter swimming paths to escape onto the hidden platform (P < 0.05), indicating that males had better performance than females. Escape latency to find hidden platform (Fig. 2.) increased in female animals when compared with male rats. There was a significant main effect of training day on escape latency [F (2.562, 35.87) = 23.22, P < 0.0001]. Overall, male rats had shorter escape latency to find the hidden platform, indicating that males had better performance than females.

#### Probe trial performance in MWM

**Fig. 3:**
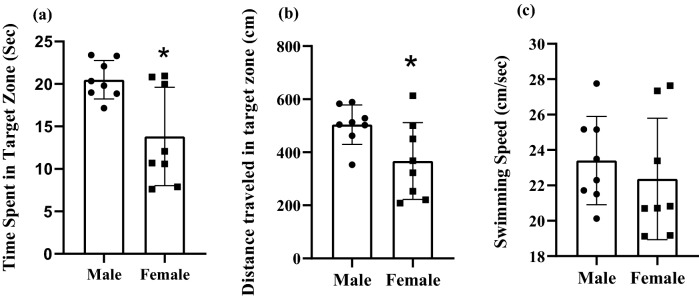
Probe trial performance of male and female rats. Female rats spent less time **a** and traveled less distance **b** in the target zone. Swimming speed was same in both male and female animals (**c**). Data presented as mean ± S.E.M. *p < 0.05

**Fig. 4:**
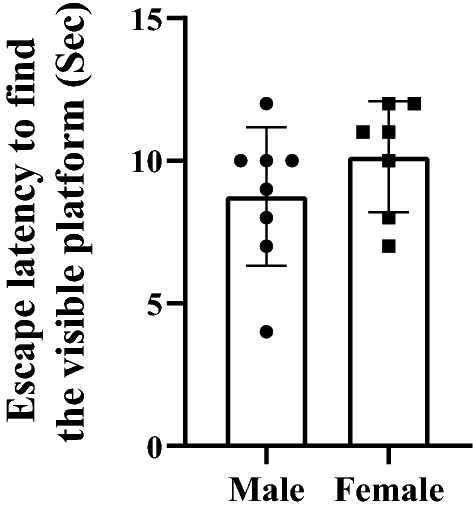
Latency to escape onto the visible platform during visible experiment. There was no visual impairment in the animals because the escape latencies to find the visible platform during visible experiment were the same in male and female rats. Data presented as mean ± S.E.M

The probe test was performed 24 h after the last training trial test on day 5 to assess the reference memory. During this test, the platform was removed and swimming speed, time spent and distance traveled in target zone of the MWM were recorded. These data are shown in Fig. 3. Female rats spent less time in the target zone in compare with male animals (female rats: 13.83 ± 2.04 s, male: 20.35 ± 1.03 s, (t_14_ = 3.03, P = 0.025, Fig. 3a). Female rats traveled less distance in the target zone in compare with male animals (t_14_ = 2.38, P = 0.03, Fig. 3b). Considering that in the probe test, the swimming speed was the same in male and female animals, it can be concluded that there are no motor disorders in these animals (t_14_ = 0.6369, P = 0.5361, Fig. 3b). In the visible experiment that took place after the probe test, all the animals were able to find the platform. The results showed that there was no visual impairment in the animals because the escape latencies to find the visible platform during visible experiment were the same in male and female rats (Fig. 4, P > 0.05).

### LTP at PP-DG synapses

**Fig. 5:**
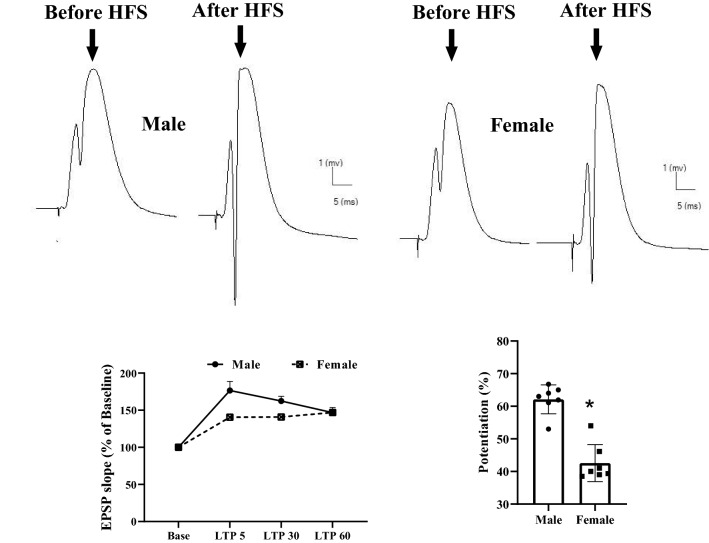
Time-dependent variations in hippocampal evoked responses to PP stimulation following an HFS. Upper panel shows representative traces of evoked field potential recording in the DG area prior to and 60 min after HFS. Male rats exhibited significantly more fEPSP slope LTP than females. Left lower panel shows fEPSP slope change (%) vs. time following HFS in both sex of rats. Bar graphs show the average fEPSP slope change (%) during 60 min post-HFS. Data are expressed as means ± SEM % of baseline. *P < 0.05

We studied LTP at PP-DG synapses in urethane-anesthetized rats to evaluate sex differences in hippocampal synaptic plasticity. Male and female rats were tested for probable differences in PP-DG LTP induction. Before and after high frequency stimulation in PP, the extracellular field potentials were recorded in the DG area. LTP was determined by examining HFS-induced changes in the fEPSP slope and PS amplitude. Representative traces of LTP recording are shown in the upper panel of Fig. 5.

#### Excitatory postsynaptic potential LTP

As shown in Fig. 5, the magnitude of fEPSP slope LTP varied with sex of the rats. Two-way repeated-measures ANOVA revealed significant effect of time- points [F (1.393, 15.32) = 15.05, P = 0.0006], significant effect of sex [F (1, 11) = 5.921, P = 0.0332], and a significant interaction of the two [F (3, 33) = 4.869, P = 0.0065] in slope of fEPSP of the granular cell of DG (Fig. 5). Post-hoc comparisons (P < 0.05) indicated that males exhibited significantly more fEPSP slope LTP than female rats. The percentage change in fEPSP slope after HFS was significantly lower in female rats than in male rats.

#### Population spike LTP

**Fig. 6:**
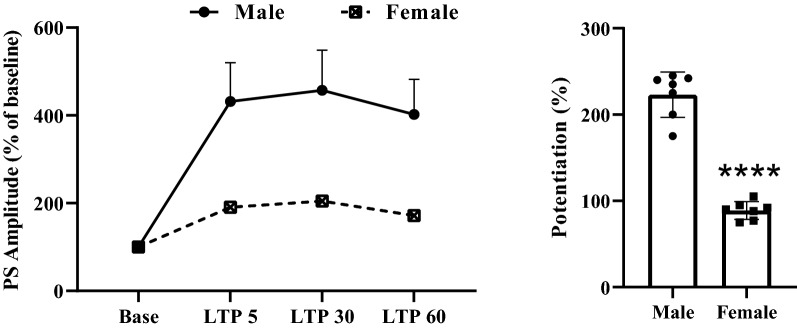
Time-dependent variations in hippocampal evoked responses to PP stimulation following an HFS. Male rats exhibited significantly more PS amplitude LTP than females. Left panel shows PS amplitude change (%) vs. time following HFS in both sex of rats. Bar graphs show the average PS amplitude change (%) during 60 min post-HFS. Data are expressed as means ± SEM % of baseline. *P < 0.0001

The sex difference in PS amplitude LTP was also evident, as shown in Fig. 6. Two-way repeated-measures ANOVA revealed significant effect of time- points [F (1.393, 15.32) = 15.05, P = 0.0006], significant effect of sex [F (1, 11) = 5.921, P = 0.0332], and a significant interaction of the two [F (3, 33) = 4.869, P = 0.0065] in amplitude of PS in the granular cell of DG (Fig. 6). Post-hoc comparisons (P < 0.0001) indicated that male rats exhibited significantly more PS amplitude LTP than female rats. The percentage change in PS amplitude after HFS was significantly lower in female rats than in male rats.

## Discussion

The present study investigated sex differences in spatial ability and synaptic plasticity. Overall, male rats had a better performance than female rats in MWM task. Sex differences in LTP were also evident in the PS amplitude and the slope of fEPSP at PP-DG synapses. Male rats exhibited significantly more PS amplitude and fEPSP slope than female rats. Our data in the current work, using Wistar rats, are consistent with most other works showing that male rats had a better performance than female rats in MWM task.

There is controversy about sex differences in synaptic plasticity. For example, sex differences in LTP induction have been reported in DG [31, 32] and in CA1 [33–35]. Furthermore, it has been reported that the LTP magnitude is not different between males and females [35, 37]. Also, study by Chen et al. Showed that amygdala LTP is higher in female mice than male mice [39]. On the other hand, it has been reported that there is no sex difference for LTP in the amygdala [40]. These different observation on sex differences in synaptic plasticity may be due to different LTP recording protocols or recording in different brain areas.

An important difference in spatial learning and memory between male and female rats appears to be that they use different learning approaches that are controlled by different areas of the brain. The preference of adult male rats is a hippocampal-dependent spatial strategy rather than a striatum-dependent response strategy. In contrast, female rats prefer a spatial strategy only when the concentration of estradiol in their bloodstream increases [41].

Sex differences in hippocampal-related behaviors suggest that there may be differences in the organization and function of hippocampal neural circuits in males and females. Some studies have reported that the male hippocampus is larger than women [42, 43], although other studies have not shown such results [44–47].

Female rats display a reduced hippocampal LTP compared to males. These changes may be related to the modulation of glutamatergic synaptic transmission and in particular the function of N-methyl-D-aspartic acid (NMDA) receptors and synaptic plasticity by estrogens [48–50]. Estradiol has been shown to affect synaptogenesis and dendritic spine density in the hippocampus in female mice. Males treated with estradiol did not show an increase in hippocampal spine density, so it can be claimed that this effect is specific to females [50]. Modulation of synaptic plasticity by estrogens may be responsible for sex differences in synaptic plasticity and lower LTP in female rats. In males, overexpression of NMDA receptors may lead to higher Ca^2+^ entry and facilitate LTP induction [51]. NMDA receptors play a main role in both LTP and spatial learning and memory [52–54].

It has also been reported that changes in the number of AMPA receptors, their subunit composition, and phosphorylation state can alter synaptic plasticity [55]. AMPA receptor function and trafficking play an important role in LTP. In females, lower trafficking of AMPA receptors in the synaptic membrane may be responsible for lower LTP [34]. The lower trafficking and insertion of AMPA receptors is due to lower activation of cGMP-dependent protein kinase (PKG) [34]. PKG inhibition prevents the insertion of AMPA receptors into membranes, resulting in a decrease in hippocampal LTP [56].

One of the reasons for the low ability of females in spatial learning and memory is lower magnitude of LTP in females, and mechanisms that reduce LTP may also be involved in low performance of females in MWM task. Because both LTP and spatial learning are disrupted by inhibition of NMDA receptors [57, 58]. Impairment in LTP and impaired spatial learning and working memory have also been reported in mice lacking the GluR-A (GluR1) AMPA receptor subunit [59].

Also, sex differences in spatial ability appear to be related to stress [60]. And female performance in the MWM may be more sensitive to stress. No sex differences in spatial performance were observed when the rats were pre-exposed to the environment [61, 62]. Pre-exposure of animals to the MWM apparatus and testing procedures before hidden platform training has been shown to reduce or eliminate sex differences in MWM performance and response to stress. Pre-exposure to MWM apparatus and testing procedures reduces stress and stress-related hormones (e.g. corticosterone) during the test, and females may have better performance in less stressful situations [63, 64]. It has also been shown that reversing the stress response by performing an adrenalectomy eliminates sex difference in MWM performance [63].

Higher thigmotaxis levels in the MWM test in females could be another cause of sex differences in spatial ability [62, 63, 65, 66]. Also, it has been shown that after MWM test, the level of thigmotaxis is positively associated with the level of corticosterone, and pre-exposure to MWM apparatus and testing procedures reduces thigmotaxis [63, 67]. The location of the platform is not the outer edge of the MWM tank,so MWM performance may be indirectly disrupted by thigmotaxis [67, 68].

It is important to understand that there is a link between estradiol levels in females and better performance in spatial skills, so that lower estradiol levels in females lead to better performance in rodents and in women [69, 70]. Since stress rises estradiol levels in females [71], this stress exposure may increase estradiol levels and lead to poorer performance in females than males.

Another possible mechanism for better performance of male rats than females is that calcium/calmodulin-dependent protein kinase kinase β (CaMKKβ) has an male-specific role in the processes of memory formation [72]. Additionally, CaMKKβ has been revealed to be essential for the activation of hippocampal cyclic AMP-responsive element binding protein (CREB) by spatial training, and to contribute to hippocampal LTP in male mice [73]. These observations suggest that hippocampal memory consolidation mechanisms are different between the sexes.

LTP has been reported to be dependent on nitric oxide synthase-1 (aNOS1) signaling in males (but not in females) [74]. Lack of nitric oxide (NO) and decreased hippocampal NOS1 expression in the female are the main causes of this sex difference [74]. However, NO-dependent LTP appears to be a secondary mechanism for LTP induction in females, but is more important in males.

## Conclusion

In summary, our results showed that there was sex difference in LTP in PP-DG synapses, which was higher in male than female rats. Therefore, our study suggests that sex associated difference in LTP may contribute to sex difference in spatial learning and memory. Lack of determination of the estrous cycle stage and the corticosterone levels may be limitations of the present study, which should be considered in future studies.

## Methods

### Animals and ethics statement

In the present study, 2-month-old male and female Wistar rats were used. A total of 15 male (8 for MWM and 7 for electrophysiology) and 15 female (8 for MWM and 7 for electrophysiology) rats were used. The housing conditions of rats were as follows: Room temperature: 22 ± 2 °C; Light–dark cycles: (12 h light‐12 h dark); The number of rats per cage: 2–3 rats; Access to food and water: free access to tap water and standard laboratory chow. The Animal Study Ethics Committee of our university approved all the experimental procedures used in this study. Also, all experimental procedures were done in accordance with the National Institutes of Health Guide for Care and Use of Laboratory Animals. Every effort was made to minimize suffering. Actions that can cause pain and distress were done in the absence of other animals in another room. Intraperitoneal injection of urethane (ethyl carbamate, 1.5 g/kg; i.p.) was used for anaesthetization of rats.

### Morris water maze (MWM) task

#### Apparatus

**Fig. 7:**
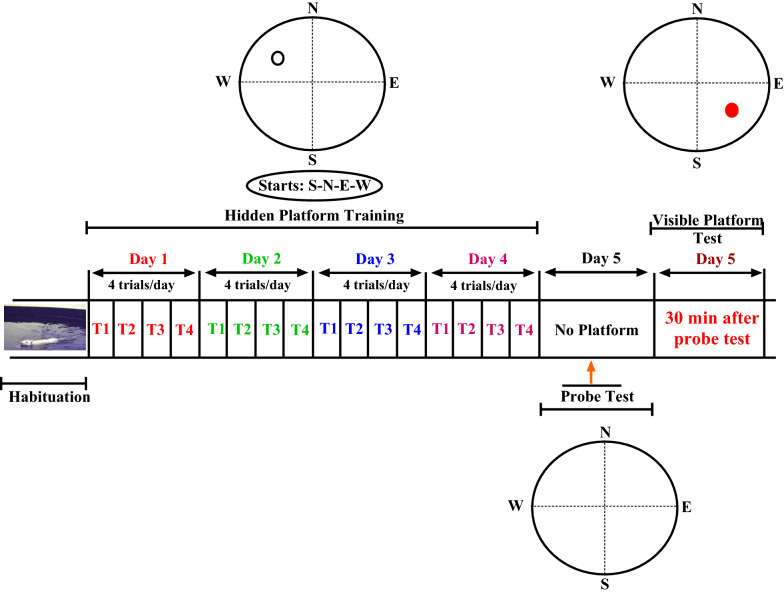
Scheme of the MWM protocol. Scheme of the Morris water maze protocol indicating habituation, hidden platform training, probe test or retention and visual test. An empty circle shows a hidden platform position. The red circle shows a visible platform position. Dashed lines represent imaginary quadrant boundaries

The MWM test, a hippocampal-dependent test, is used to assess spatial learning and memory in rodents [75–78]. The main advantage of MWM task is the distinction between spatial conditions (hidden platform) and non-spatial conditions (visible platform). Furthermore, the MWM test environment decreases odor trail interference. The MWM device consisted of a black circular pool with a diameter of 155 cm and a height of 60 cm, which was filled with water at a temperature of 22 ± 1 °C to a depth of 35 cm. The pool was divided into four equal quadrants. An invisible platform with a diameter of 10 cm, made of clear Plexiglas, was placed in the center of the eastern quarter as the target quadrant at a distance of 2 cm below the water surface. A video-computer tracking system (CCD camera, Panasonic Inc., Japan) was used to record the rats' swim path for further analysis (EthoVision software XT7, Netherland). Large posters were used on the wall of the room as visual cues. Scheme of the MWM protocol is shown in Fig. 7.

#### Habituation

In order to adapt the rats to the MWM test, one day before the starting of training, the rats swam for one minute in a tank without a platform.

#### Hidden platform training

The training sessions were done according to our earlier works [75, 77, 79–81]. Briefly, the training session was conducted for four consecutive days (one block of 4 trials per day). Each trial began by putting the rat in the middle of one of the four quadrants. The swimming time to find the hidden platform was 90 s. If the animal did not find the hidden platform during this period, it was manually transferred to the platform by the investigator. The time between the two consecutive trials was 10 min. To assess acquisition of the MWM task during training days, escape latency (i.e., time to reach the platform) and swimming distance were recorded. The daily average of all trials from day 1 to day 4 was used in our analysis.

#### Spatial reference memory (Probe test or retention)

The spatial probe test was performed one day after the last training session. In the spatial probe test, rats swam for 60 s in a pool without platform. The animals were released into the water in the western quadrant (ie, exactly opposite from where the platform was placed in the training sessions). A video-computer tracking system was used to record the distance traveled and time spent in target zone as well as swimming speed of rats (to evaluate the motor activity).

#### Visual test

Thirty minutes after the probe trial, visual test was performed. In the visual test, the platform was covered with bright color aluminum foil and placed in a different zone above the water surface. In the visual test, rats swam for 60 s to find the visible platform in order to test their visual ability. All experiments were performed between 12:00 and 14:00.

### Surgical procedure, electrophysiological recording and LTP induction

The procedures used here were done according to our previous works [82–84]. Briefly, after urethane anesthesia (1.5 g/kg, i.p), the rats were placed in a stereotaxic device for electrode implantation surgery and field potential recording. Using a heating pad, the temperature of the animals was kept constant at 36.5 ± 0.5 °C. After the skull is exposed, small holes were drilled in the skull, and then two bipolar stimulating and recording electrodes were implanted in the right cerebral hemisphere. The electrodes were made of Teflon coated stainless steel (125 µm diameter, Advent Co., UK). The coordinates for electrode placement were as follows (according to the Paxinos and Watson atlas of the rat brain [82, 85]): Stimulating electrode in the PP [:AP: − 8.1 mm from bregma; ML: + 4.3 mm from midline; DV: 3.2 mm from the skull surface], and recording electrode in the DG granular cell layer [AP: − 3.8 mm from bregma; ML: + 2.3 mm from midline; DV: 2.7–3.2 mm from the skull surface]. To minimize damage to the brain, the electrodes were lowered very slowly (0.2 mm/min) from cortex to the hippocampus.

We obtained input–output profile by PP stimulation to determine the intensity of stimulus used in each animal (40% maximal population spike). Through constant current isolation units (A365 WPI), single 0.1 ms biphasic square wave pulses were delivered at a frequency of 0.1 Hz. The field potential recordings evoked by stimulation of the PP were recorded extracellularly in the DG area. Test stimuli were delivered to the PP every 10 s. Electrodes were positioned to elicit the maximum amplitude of PS and fEPSP. After recording the steady-state baseline response for 40 min, LTP was induced using the 400 Hz HFS (including10 bursts of 20 stimuli, 0.2 ms stimulus duration, 10 s interburst interval). DG granular neurons were recorded at 5, 30, and 60 min post-HFS stimulation to determine any change in the both fEPSP and PS. An average of 10 consecutive recordings were made at 10 s stimulus interval for each time point [83, 86, 87]. For stimulations, stimulus parameters were defined in the software at the beginning and prior delivery to PP, then sent via a data acquisition board connected to a constant current isolator unit (A365 WPI, USA). The evoked responses in the DG region after passing through a preamplifier, were amplified (1000 ×) (Differential amplifier DAM 80 WPI, USA), and were filtered (band pass 1 Hz to 3 kHz). These responses were digitized at a sampling rate of 10 kHz, and were observable on a computer monitor. These responses were stored in a high-speed data storage device for later offline analysis.

### Measurement of evoked potentials

PS and fEPSP are two components of the evoked recording in the DG. In our work, PS amplitude and fEPSP slope were measured. The PS amplitude was measured from the peak of the first positive deflection of the evoked potential to the peak of the following negative potential. The fEPSP slope function was measured as the slope of the line connecting the start of the first positive deflection of the evoked potential with the peak of the second positive deflection of the evoked potential. The stimulation intensity was adjusted to evoke potentials which comprised 40% of the maximal population spike amplitude, defined by means of an input/output curve [82, 83].

### Statistical analysis

GraphPad Prism® 8.0.2 software (San Diego, CA, USA) was used for statistical analysis. Data were presented as means ± standard error mean (S.E.M.). The data of the training trials in MWM were analyzed using a two-way analysis of variance (ANOVA) (followed by Bonferoni post hoc) with days as repeated measures factor and treatments as between subjects’ factor. Student t-test was used for statistical analyses of probe and visibility trial data. Two-way repeated-measures ANOVA followed by Bonferroni post hoc was used for analysis of the LTP data. Student t test was used when only two values were compared. P values below 0.05 were considered statistically significant. The following formula was used to determine LTP values:

$$ {\text{LTP}} = \frac{{{\text{the EPSP or PS value after HFS induction }} \times 100\% }}{{{\text{the average EPSP or PS at baseline}}}}  $$

## Data Availability

The data are available for any scientific use with kind permission.
